# Otology

**Published:** 1902-12

**Authors:** W. H. Harsant


### OTOLOGY.

The **treatment of obstinate strictures of the Eustachian tube by electrolysis** has been frequently attempted of late years, and the subject is well dealt with by Dr. Norval H. Pierce in a recent number of the *Archives of Otology.*[1]

He alludes to the fact that Cumberbatch and Steavenson published a series of seven cases so treated in 1888,[2] and that

[1] *Arch. Otol.*, 1902, xxxi. 289. [2] *Lancet*, 1888, ii. 1014.

Robert Newman in a paper published in 1898[1] also brought forward some work in this direction. Fresh impetus was given to the subject by a paper published by Duel in 1897.[2] By substituting a gold bougie for the more or less cumbersome electrodes of his predecessors, Duel greatly improved instrumentation.

Dr. Pierce has subjected twenty-one cases during the past twelve months to this mode of treatment. The electric current was taken from a circuit of 110 volts, and led through a Victor shunt-controller. From 15 to 30 volts were necessary to obtain from two to five milliampères with the negative pole attached to the Eustachian bougie, while the positive pole in contact with the sponge electrode was held in the hand opposite to the tube operated on. The gold electric bougies made by Meyrowitz were employed. Contacts lasted from three to ten minutes. Lengths of contacts and strength of current varied according to the electrical resistance of the patient and the resistance offered to the passage of the electrode in the tube and the subjective sensations of the patient. Both the plain rubber catheter and the silver catheter were used. The silver catheter insulated by means of rubber tissue is more easily kept clean and more readily adapted, by bending, to the peculiar topography of each case, than the rubber catheter. The procedure was nearly always carried on under cocaine, all active inflammatory processes having been first allayed, and the strictest cleanliness observed.

Ten cases were in the class of oto-sclerosis, or rarefaction of the labyrinthine capsule. Eight were catarrhal. Of the remaining two, one was due to disease of the nervo-muscular apparatus of the tube, the other was one of almost complete obliteration of the membranous portion, due to syphilis.

All the cases had been treated by other methods before treatment was begun with the electrical bougie. Before beginning with the electrical treatment, all the tubes were explored by means of a celluloid bougie. In six of the cases of oto-sclerosis the bougie was arrested at about the isthmus. In two of these six cases, the electrical bougie passed through the osseous portion after the fourth sèance. They were all treated for two months, at intervals of a week, with catheterization every other day. In none was audition improved to a noteworthy extent. In six of the eight cases of catarrhal disease, the bougie was arrested from 4 to 23 mm. from the pharyngeal opening. In five of them, after four months' treatment, the electrical bougie failed to pass further than the isthmus, or a few millimetres beyond. In two cases in which the bougie passed directly into the middle ear, there were extensive adhesions between the tympanic membrane and the promontory. In none of these cases was there any improvement—that is, further than the improvement gained by the other methods of treatment previously pursued, nor was

[1] *Med. Rec.*, 1898, liv. 870.

[2] *N.Y. Eye & Ear Inf. Rep.*, 1897; quoted by Pierce, *loc. cit.*

there any alteration in the adhesions. The case of syphilitic stenosis remained unchanged. In only two of the twenty cases could any results be ascribed to the electrical bougie. These were both cases of subacute disease with recurrent attacks of defective audition and tinnitus with diminishing intervals. In these cases there were, in all probability, soft infiltrations in the membrano-cartilaginous tube near the isthmus. In both of them the benefit to audition and the subjective symptoms was marked, immediate, and lasting, and these results were obtained after the usual methods of injection, the catheter, inflation, and massage, had ceased to benefit.

Occasionally, in other cases, marked improvement to audition was observed immediately after the application of the electrode, but this cannot be ascribed to the influence of the electricity, inasmuch as we occasionally observe as great improvement following the use of the ordinary celluloid bougie. The explanation of this phenomenon is difficult, but probably takes place by way of reflex stimulation of the nervous apparatus of the ear.

Dr. Pierce found the electrical bougie rather painful. The use of the wire bougie in the Eustachian tube is not without danger. Several instances of acute otitis media have followed its use. There have been instances where the electrical bougie has broken off in the tube, and owing to the proximity of the internal carotid artery other accidents may occur from the accidental wounding of a blood-vessel. He comes to the following conclusions:—(1) In oto-sclerosis, electrolysis is useless; (2) In the great majority of cases of catarrhal disease, it has no advantages over other methods of treatment; (3) In a certain few cases where there is probably a soft exudate near the isthmus, it may be regarded as of some value.

W. H. Harsant.

